# Lamin is essential for nuclear localization of the GPI synthesis enzyme PIG-B and GPI-anchored protein production in *Drosophila*

**DOI:** 10.1242/jcs.238527

**Published:** 2020-03-26

**Authors:** Miki Yamamoto-Hino, Kohei Kawaguchi, Masaya Ono, Kazuhiro Furukawa, Satoshi Goto

**Affiliations:** 1 Department of Life Science, College of Science, Rikkyo University, Toshima-ku, Tokyo 171-8501, Japan; 2 Department of Clinical Proteomics, National Cancer Center Hospital, Chu-o-ku, Tokyo 104-0045, Japan; 3 Department of Chemistry, Faculty of Science, Niigata University, Niigata 950-2181, Japan

**Keywords:** Glycosylphosphatidylinositol, Nuclear envelope, Endoplasmic reticulum, PIG, Lamin, Organelle zone

## Abstract

Membrane lipid biosynthesis is a complex process that occurs in various intracellular compartments. In *Drosophila*, phosphatidylinositol glycan-B (PIG-B), which catalyzes addition of the third mannose in glycosylphosphatidylinositol (GPI), localizes to the nuclear envelope (NE). Although this NE localization is essential for *Drosophila* development, the underlying molecular mechanism remains unknown. To elucidate this mechanism, we identified PIG-B-interacting proteins by performing immunoprecipitation followed by proteomic analysis. We then examined which of these proteins are required for the NE localization of PIG-B. Knockdown of Lamin Dm0, a B-type lamin, led to mislocalization of PIG-B from the NE to the endoplasmic reticulum. Lamin Dm0 associated with PIG-B at the inner nuclear membrane, a process that required the tail domain of Lamin Dm0. Furthermore, GPI moieties were distributed abnormally in the *Lamin Dm0* mutant. These data indicate that Lamin Dm0 is involved in the NE localization of PIG-B and is required for proper GPI-anchor modification of proteins.

## Abstract (precis)

**Highlighted Article:** Lamin plays a role in post-translational modification of plasma membrane proteins by tethering the GPI modification enzyme PIG-B to the inner nuclear membrane.

## INTRODUCTION

Synthesis, remodeling and degradation of membrane lipids occur in various cellular compartments including the endoplasmic reticulum (ER), mitochondria, Golgi complex and endosomes. The glycolipid glycosylphosphatidylinositol (GPI) functions as a membrane anchor for multiple cellular proteins. GPI-anchored proteins play important roles in various biological processes such as development, immunity, neural functions and physiological homeostasis.

GPI is synthesized from phosphatidylinositol via a multi-step reaction catalyzed by phosphatidylinositol glycan (PIG) enzymes (Kinoshita, 2016). Seventeen PIG enzymes have been identified in mammals (Kinoshita, 2014). Exogenously expressed PIG enzymes localize throughout the ER in cultured mammalian cells (Kang et al., 2005; Maeda et al., 2001; Murakami et al., 2005, 2003; Nakamura et al., 1997; Taron et al., 2004; Vainauskas et al., 2002; Watanabe et al., 1996). However, in mammalian cells (Vidugiriene et al., 1999) and yeast (Castillon et al., 2009; Lopez et al., 2019; Muñiz et al., 2001), some types of GPI synthesis, such as de-N-acetylation of GlcNAc-PI by an endogenous PIG enzyme and loading of GPI-anchoring of proteins into transport vesicles, respectively, occur in a subcompartment of the ER. This subcompartmentalization of GPI-anchored protein synthesis and sorting is conserved in *Drosophila*. We previously reported that most PIG enzymes localize to the ER, but *Drosophila* PIG-B, which catalyzes addition of the third mannose in GPI (Takahashi et al., 1996), localizes to the nuclear envelope (NE) (Yamamoto-Hino et al., 2018). We generated an ER-localized PIG-B form, called PIG-B[ER], to determine whether the NE localization of PIG-B is functionally important. Whereas expression of wild-type PIG-B completely rescues the lethality of the *PIG-B* mutant (*PIG-B*^13^), expression of PIG-B[ER] does not. Moreover, PIG-B[ER] is preferentially degraded by lysosomes. These data suggest that the NE localization of PIG-B is essential for its function and stability. In addition, we found that translation of GPI-anchored proteins and attachment of GPI occur in the region of the ER proximal to the NE. Thus, we propose that the NE and proximal ER form the GPI modification zone (one of the ‘organelle zones’), which is defined as a functional platform within an organelle or spanning multiple organelles that allows efficient synthesis of GPI-anchored proteins. It is important to elucidate the mechanism underlying the NE localization of PIG-B in order to understand how this zone forms.

The NE separates the nuclear and cytoplasmic compartments of eukaryotic cells and consists of the outer and inner membranes joined at sites that are occupied by the nuclear pore complex (NPC). The outer nuclear membrane (ONM) is continuous with the ER and is covered with ribosomes. The inner nuclear membrane (INM) is undercoated by the nuclear lamina, the main components of which are lamins. Lamins are type V intermediate filaments that form a meshwork of filaments at the nuclear periphery (de Leeuw et al., 2018; Gruenbaum and Foisner, 2015). These proteins are divided into A- and B-types based on their biochemical properties, expression patterns, and behaviors during mitosis. In mammals, there are three lamin genes, one A-type (*LMNA*) and two B-types (*LMNB1* and *LMNB2*). *LMNA* is alternatively spliced to produce two major isoforms called Lamin A and C. *Drosophila* cells express A/C- and B-type lamins called Lamin C and Lamin Dm0, respectively (Melcer et al., 2007). These proteins have a short N-terminal head domain, a long α-helical coiled-coil rod domain, and a tail domain containing a nuclear localization signal and an immunoglobulin fold. The rod domain mediates lamin dimerization, whereas the head and tail domains mediate head-to-tail polymer assembly. Lamins bind to many known INM and chromatin proteins to exert their functions such as maintenance of nuclear morphology, positioning of the nucleus, chromatin organization and gene expression. However, lamins have not been reported to function in post-translational modification of secretory and membrane proteins without affecting transcription.

Here, we show that Lamin Dm0, but not Lamin C, associated with PIG-B. Depletion of Lamin Dm0 resulted in mislocalization and decreased expression of PIG-B in cultured cells and *Drosophila*, suggesting that Lamin Dm0 is required for the NE localization and stability of PIG-B. Furthermore, GPI moieties were not observed at the plasma membrane in the *Lamin Dm0* mutant. Taken together, these findings demonstrate that Lamin Dm0 is necessary for tethering of PIG-B at the NE and proper GPI synthesis.

## RESULTS

### Lamin Dm0 is required for the NE localization of PIG-B

To clarify the mechanism underlying the NE localization of PIG-B, we first attempted to identify PIG-B-interacting proteins in S2 cells. We generated S2 cell lines that stably expressed Flag-tagged PIG-B (PIG-B-Flag) and immunoprecipitated PIG-B-Flag and its interacting proteins using an anti-Flag antibody following crosslinking with formamide. S2 cells not expressing PIG-B-Flag served as a control. Immunoprecipitated proteins were analyzed by two-dimensional image-converted analysis of liquid chromatography and mass spectrometry (2DICAL) (Ono et al., 2018, 2006). A total of 1883 independent mass spectrometry peaks were detected and 388 *Drosophila* proteins were assigned. Among these, the levels of 107 proteins were more than 2-fold higher in the PIG-B-Flag immunoprecipitate than in the control (>2-fold difference in intensity compared with the control) (Table S1).

**Fig. 1.**
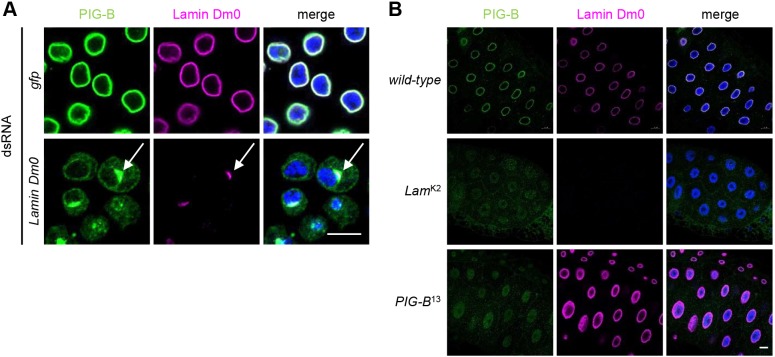
**Lamin Dm0 is required for the NE localization of PIG-B.** (A,B) Immunofluorescence analysis of PIG-B in GFP- or Lamin Dm0-knockdown S2 cells (A) and salivary glands of the wild-type, *Lamin Dm0* mutant (*Lam*^K2^) and *PIG-B* mutant (*PIG-B*^13^) (B). Cells and salivary glands were stained with an anti-PIG-B antibody (green), an anti-Lamin Dm0 antibody (magenta) and DAPI (blue). White arrows indicate strong co-localization of Lamin Dm0 and PIG-B. Scale bars: 10 µm in A; 20 µm in B.

PIG-B localizes to the NE; therefore, we first chose Lamin Dm0, Torsin, Lamin-B receptor (LBR), Krueppel homolog 2 (Kr-h2) and Otefin (Ote) among the hits because they localize to the NE. Then, we selected SERCA, Surfeit locus protein 4 homolog (Surf4; hereafter referred to as Surfeit 4) and Jagunal (Jagn), which interact with the five proteins selected from the hit list (Table 1). To test which of these are required for the NE localization of PIG-B, we knocked down the selected genes in S2 cells and examined the localization of PIG-B. Knockdown of Lamin Dm0 resulted in mislocalization of PIG-B to the ER (Fig. 1A). Moreover, PIG-B strongly associated with residual Lamin Dm0 in the NE (arrows in Fig. 1A). Punctate signals derived from PIG-B were detected in the cytosol of S2 cells in which Kr-h2, Otefin, Jagunal and Torsin were knocked down (Fig. S1); some of these punctate signals co-localized with Lamin Dm0 (Fig. S1, insets). These data suggest that Lamin Dm0 is required for the NE localization of PIG-B, probably because of their direct or indirect interaction.

**Table 1.**
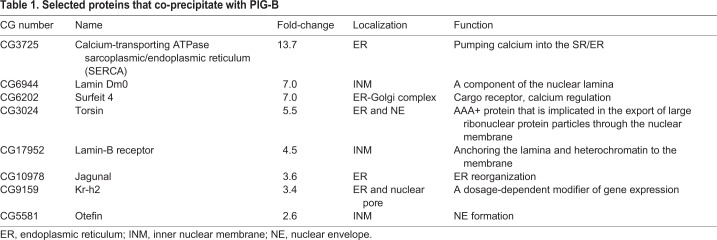
**Selected proteins that co-precipitate with PIG-B**

We next investigated whether PIG-B was mislocalized in the *Drosophila Lamin Dm0* (*Lam*^K2^) mutant, in which a frame shift occurs after amino acid 153 of Lamin Dm0 due to a nucleotide insertion (Patterson et al., 2004). As *Lam*^K2^ is larval lethal, we analyzed PIG-B localization at the larval stage. PIG-B was detected in the NE in salivary glands of wild-type late third instar larvae (control) (Fig. 1B); however, PIG-B was hardly detected in *Lam*^K2^ larvae. The weak signals observed throughout the nucleoplasm of *Lam*^K2^ might be nonspecific because they were also detected in salivary glands of the *PIG-B* mutant (*PIG-B*^13^), which lacks the translational start codon (Yamamoto-Hino et al., 2018).

We previously reported that ER-localized PIG-B is degraded by lysosomes (Yamamoto-Hino et al., 2018). Based on this previous result and the finding that PIG-B was hardly detected in late third instar larvae of *Lam*^K2^ (Fig. 1B), we hypothesized that loss of PIG-B in *Lam*^K2^ may be caused by degradation of mislocalized PIG-B during larval development. To examine whether Lamin Dm0 is required for the NE localization of PIG-B, we observed the localization of PIG-B at the early stage of third instar larvae of *Lam*^K2^. PIG-B was distributed throughout salivary gland cells, and this localization may correspond with the ER (Fig. S2A). An anti-PIG-B antibody also stained the nucleoplasm in early third instar larvae. However, this staining may be nonspecific because it was also detected in early *PIG-B* mutant larvae (Fig. S2A). In addition, we stained peripodial cells, which are large squamous epithelial cells, to more easily examine the localization of PIG-B. Although PIG-B and Lamin Dm0 exhibited an elliptical localization in the wild-type, in the cytoplasm of *Lam*^K2^ PIG-B was detected as a mesh-like structure, typical of the ER (Fig. S2B). These data show that Lamin Dm0 is responsible for the NE localization of PIG-B *in vivo*.

### Lamin Dm0 associates proximally with PIG-B

**Fig. 2.**
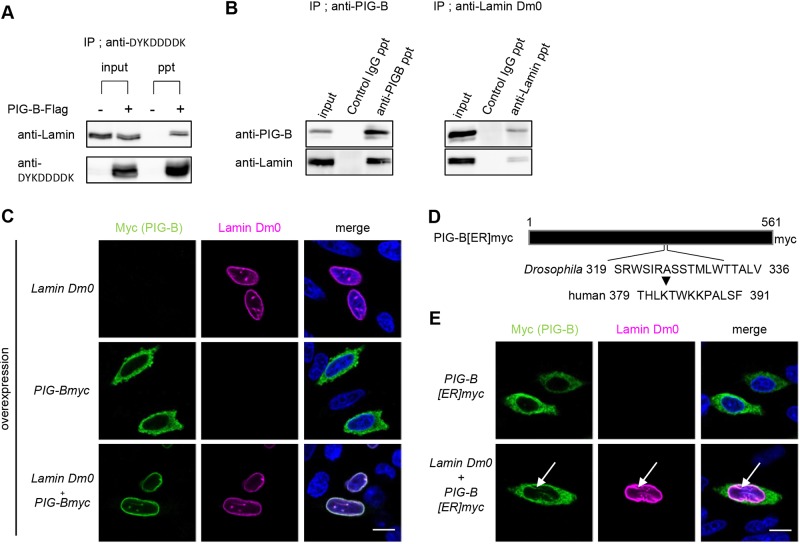
**Lamin Dm0 associates proximally with PIG-B.** (A) Immunoprecipitation experiments using lysates of S2 cells expressing or not expressing (as a control) PIG-B-Flag with anti-DYKDDDDK antibody-conjugated magnetic beads. Immunoblot analysis was carried out with anti-Lamin Dm0 and anti-DYKDDDDK antibodies. (B) Co-immunoprecipitation experiments using S2 cell lysates with anti-PIG-B (left) and anti-Lamin Dm0 (right) antibodies. Normal mouse IgG was used as a control. Immunoblot analysis was carried out with anti-PIG-B (upper) and anti-Lamin Dm0 (lower) antibodies. Input, input; ppt, precipitates. (C) Immunofluorescence analysis of CHO cells expressing Lamin Dm0 and/or myc-tagged PIG-B. Cells were stained with an anti-myc antibody (green), an anti-Lamin Dm0 antibody (magenta) and DAPI (blue). (D) Schematic of ER-localized myc-tagged PIG-B in which residues 319–336 were replaced with the corresponding portion of human PIGB. (E) Immunofluorescence analysis of CHO cells expressing ER-localized myc-tagged PIG-B (PIG-B[ER]myc) alone or together with Lamin Dm0. Cells were stained with an anti-myc antibody (green), an anti-Lamin Dm0 antibody (magenta) and DAPI (blue). White arrows indicate co-localization of PIG-B[ER]myc and Lamin Dm0. Scale bars: 10 µm.

To examine the association of Lamin Dm0 and PIG-B, we expressed PIG-B-Flag in S2 cells and subjected cell lysates to immunoprecipitation with an anti-Flag antibody. S2 cells not expressing PIG-B-Flag served as a control. Immunoblotting with an anti-Lamin Dm0 antibody revealed that Lamin Dm0 co-precipitated with PIG-B-Flag, but this co-precipitation was not detected in control cells (Fig. 2A). We further tested whether endogenous PIG-B and Lamin Dm0 associated with each other in S2 cells. Lamin Dm0 co-precipitated with PIG-B (Fig. 2B, left) and vice versa (Fig. 2B, right).

We next examined whether Lamin Dm0 is sufficient for localization of PIG-B to the NE. Myc-tagged PIG-B localized to the cytosol when expressed in CHO cells (Fig. 2C). This suggests that no protein tethers PIG-B to the NE in these cells. However, expression of Lamin Dm0, which localized to the NE in CHO cells, resulted in the NE localization of PIG-B (Fig. 2C). This NE localization could not be due to a defect in the ER because overexpression of Lamin Dm0 did not affect ER morphology, as detected using an anti-KDEL antibody (Fig. S3A). These results support the notion that Lamin Dm0 is sufficient for NE localization of PIG-B.

We previously generated an ER-localized form of PIG-B (called PIG-B[ER]) by replacing the NE localization sequences (residues 319–336) of PIG-B with the corresponding portion of human PIGB (Fig. 2D) (Yamamoto-Hino et al., 2018). Myc-tagged PIG-B[ER] localized to the ER in CHO cells similar to myc-tagged wild-type PIG-B (Fig. 2E, upper). We expressed PIG-B[ER] and Lamin Dm0 in CHO cells. Although Lamin Dm0 localized to the NE, almost all PIG-B[ER] remained in the ER (Fig. 2E, lower). This result suggests that the replaced amino acid sequence is one of the sites via which PIG-B interacts with Lamin Dm0. Notably, a portion of PIG-B[ER] co-localized with Lamin Dm0 in the nucleoplasm (arrows, Fig. 2E, lower). This co-localization was more apparent in CHO cells that strongly expressed Lamin Dm0 (Fig. S3B). Approximately 95% of cells expressing both Lamin Dm0 and PIG-B[ER] showed co-localization in the nucleoplasm (25 of 26 cells), whereas only 17% of cells expressing solely PIG-B[ER] exhibited nucleoplasmic localization of PIG-B[ER] (four of 23 cells). In addition, we previously reported that NE localization requires other regions of PIG-B that are unchanged in PIG-B[ER] (Yamamoto-Hino et al., 2018). Therefore, multiple regions of PIG-B, including residues 319–336, may contribute to its association with Lamin Dm0.

### PIG-B localizes to the INM

**Fig. 3.**
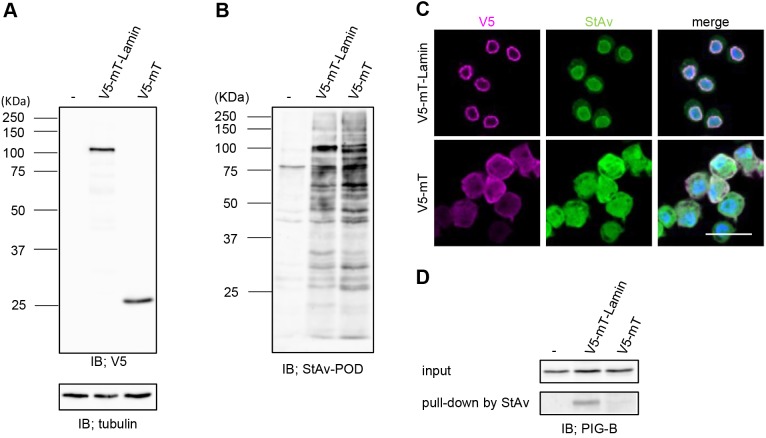
**Proximity-dependent labeling assay showing that PIG-B localizes to the INM.** (A,B) Immunoblot analysis of S2 cells transfected with the empty vector (−) or expressing V5-mT-Lamin or V5-mT using anti-V5 and anti-α-tubulin antibodies (A) and streptavidin-POD (B). (C) Localizations of V5-mT-Lamin and V5-mT expressed in S2 cells and biotinylated proteins. Cells were stained with an anti-V5 antibody (magenta). Biotinylated proteins were visualized using Alexa Fluor 488-conjugated streptavidin (green). Nuclei were stained with DAPI (blue). Scale bar: 20 µm. (D) Immunoblot analysis of 0.5% of cell lysates used for immunoprecipitation (input) and precipitates (pull-down) obtained with streptavidin magnetic beads from S2 cells expressing V5-mT-Lamin or V5-mT using an anti-PIG-B antibody.

The association between PIG-B and Lamin Dm0 indicates that PIG-B is targeted to the INM because lamins undercoat this membrane. To investigate this possibility, we performed a proximity-dependent labeling assay using the biotin ligase miniTurboID (Branon et al., 2018). We established S2 cell lines harboring transgenes encoding lamin-conjugated miniTurboID tagged with V5 (V5-mT-Lamin) or non-conjugated miniTurboID tagged with V5 (V5-mT). Expression of both forms of miniTurboID was induced by Cu^2+^ (Fig. 3A). Incubation with biotin for 6 h induced biotinylation in a miniTurboID-dependent manner (Fig. 3B). Fluorescence staining with an anti-V5 antibody revealed that V5-mT-Lamin and V5-mT localized to the NE and cytosol, respectively (Fig. 3C, magenta). Biotinylated proteins detected by Alexa Fluor 488-labeled streptavidin localized to the nucleus in V5-mT-Lamin-expressing cells, but localized throughout V5-mT-expressing cells (Fig. 3C, green). Biotinylated proteins in cell lysates were captured by streptavidin magnetic beads and subjected to 2DICAL to examine whether lamin-binding proteins were enriched in the biotinylated fraction from V5-mT-Lamin-expressing cells. We measured control (V5-mT) and V5-mT-Lamin samples in triplicate and identified 27 proteins showing >2-fold enrichment and *P*-values <0.05 (Table S2). The most enriched protein was Bicaudal D (BicD) (21.8-fold), which binds Lamin Dm0 (Stuurman et al., 1999). *Drosophila* emerin homolog Otefin and Lamin-B receptor, both of which are well-known lamin-binding proteins, were enriched by 10.5- and 4.8-fold, respectively (Goldberg et al., 1998; Wagner et al., 2004). In addition, Uniprot identified more than 50% of enriched proteins as being localized to the nucleus (https://www.uniprot.org/). From these results, we conclude that our proximity-dependent labeling experiment works well. Then, we performed immunoblotting with an anti-PIG-B antibody. PIG-B was detected in the precipitate from V5-mT-Lamin-expressing cells, but not in that from S2 cells or control V5-mT-expressing cells (Fig. 3D). These results suggest that PIG-B localizes to the INM.

### Lamin C is not required for the NE localization of PIG-B

**Fig. 4.**
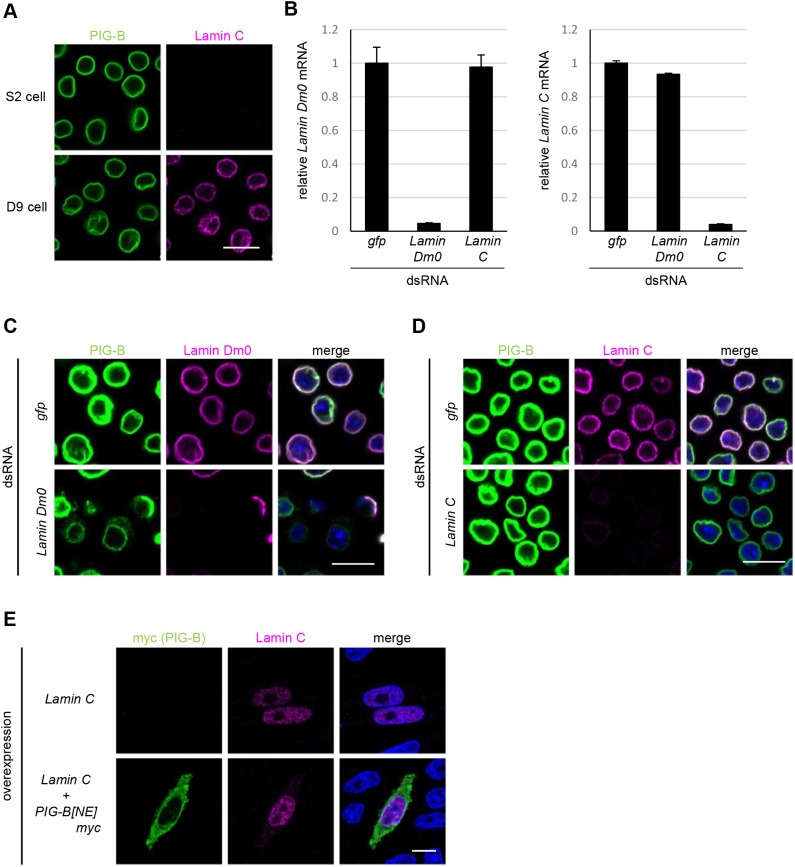
**Lamin C is not required for the NE localization of PIG-B.** (A) Immunofluorescence analysis of S2 and D9 cells stained with anti-PIG-B (green) and anti-Lamin C (magenta) antibodies. (B) mRNA levels of Lamin Dm0 (left) and Lamin C (right) in GFP-, Lamin Dm0- and Lamin C-knockdown D9 cells. Data are mean±s.e.m. of triplicate data. (C) Localizations of PIG-B and Lamin Dm0 in GFP- and Lamin Dm0-knockdown D9 cells. Cells were stained with an anti-PIG-B antibody (green), an anti-Lamin Dm0 antibody (magenta) and DAPI (blue). (D) Localizations of PIG-B and Lamin C in GFP- and Lamin C-knockdown D9 cells. Cells were stained with an anti-PIG-B antibody (green), an anti-Lamin C antibody (magenta) and DAPI (blue). (E) Immunofluorescence analysis of CHO cells expressing Lamin C alone or together with myc-tagged PIG-B. Cells were stained with an anti-myc antibody (green), an anti-Lamin C antibody (magenta) and DAPI (blue). Scale bars: 10 µm.

*Drosophila melanogaster* expresses another lamin gene encoding Lamin C. We investigated whether Lamin C was also involved in the NE localization of PIG-B. Lamin C was not expressed in S2 cells, but was detected in the nucleus of D9 cells (Fig. 4A). We carefully chose RNAi target regions that lack significant homology between the two lamin genes and performed gene knockdown in D9 cells. The specificity and efficiency of RNAi were confirmed by qPCR and immunostaining (Fig. 4B–D). Knockdown of Lamin Dm0 resulted in mislocalization of PIG-B to the ER in D9 cells, the same as in S2 cells (Fig. 4C). However, knockdown of Lamin C did not affect the NE localization of PIG-B (Fig. 4D). These data suggest that Lamin C is not required for the NE localization of PIG-B. Next, we examined the localization of PIG-B in CHO cells in which *Drosophila* Lamin C was co-expressed. Lamin C lacks a CaaX motif in its C-terminus (Melcer et al., 2007), which is farnesylated and necessary for tethering of lamins to the peripheral lamina, and was therefore distributed throughout the nucleoplasm (Fig. 4E). The localization of PIG-B to the cytoplasm did not change when Lamin C was co-expressed in CHO cells. Taken together, these findings demonstrate that Lamin C is not involved in the NE localization of PIG-B.

### The tail domain of Lamin Dm0 is required for tethering of PIG-B to the INM

**Fig. 5.**
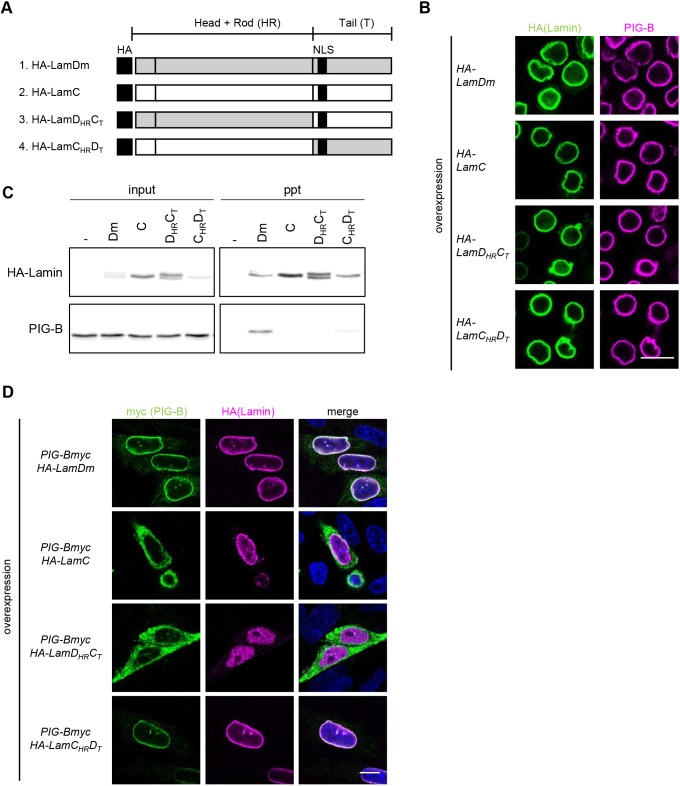
**The tail domain of Lamin Dm0 is required for tethering of PIG-B to the INM.** (A) Schematic (modified from Uchino et al., 2017) of HA-tagged chimeric lamins used in this study. (B) Immunofluorescence analysis of S2 cells expressing HA-tagged chimeric lamins. Cells were stained with anti-HA (green) and anti-PIG-B (magenta) antibodies. (C) Co-immunoprecipitation experiments using lysates of S2 cells transfected with the empty vector (–) or expressing HA-tagged chimeric lamins with anti-HA antibody-conjugated magnetic beads. Immunoblot analysis of 0.5% of cell lysates used for immunoprecipitation (input) and precipitates (ppt) was carried out with anti-HA (upper) and anti-PIG-B (lower) antibodies. (D) Immunofluorescence analysis of CHO cells co-expressing myc-tagged PIG-B and HA-tagged chimeric lamins. Cells were stained with an anti-myc antibody (green), an anti-HA antibody (magenta) and DAPI (blue). Scale bars: 10 µm.

To identify the Lamin Dm0 domain required for association with PIG-B, we expressed hemagglutinin (HA)-tagged chimeric proteins of Lamin Dm0 and Lamin C (Fig. 5A) (Uchino et al., 2017) in S2 cells and investigated whether they interact with endogenous PIG-B. All chimeric proteins localized to the rim of the INM (Fig. 5B). Cell lysates were subjected to immunoprecipitation with an anti-HA antibody followed by immunoblotting using anti-PIG-B and anti-HA antibodies. The interaction profiles were investigated. HA-tagged Lamin Dm0 (HA-LamDm) bound to PIG-B, but HA-tagged Lamin C (referred to as LamC_HR_C_T_ by Uchino et al., 2017) did not (Fig. 5C). A weak but obvious interaction was observed between PIG-B and HA-LamC_HR_D_T_, in which the head and rod domains were Lamin C-type and the tail domain was Lamin Dm0-type. However, no interaction was detected between PIG-B and HA-LamD_HR_C_T_, in which the head and rod domains were Lamin Dm0-type and the tail domain was Lamin C-type.

Furthermore, the interactions of these chimeric proteins with myc-tagged PIG-B were examined in CHO cells. As shown above, PIG-B localized to the NE or ER when expressed with Lamin Dm0 (HA-LamDm) or Lamin C (HA-LamC), respectively (Figs 2C, 4E and 5D). PIG-B re-localized from the ER to the NE when HA-LamC_HR_D_T_ was expressed, but remained in the ER when HA-LamD_HR_C_T_ was expressed (Fig. 5D). These results were consistent with those obtained in co-immunoprecipitation experiments. Taken together, these findings demonstrate that the tail domain of Lamin Dm0 is required for the association with PIG-B.

### The *Drosophila Lamin Dm0* mutant exhibits a defect in GPI-anchored proteins

**Fig. 6.**
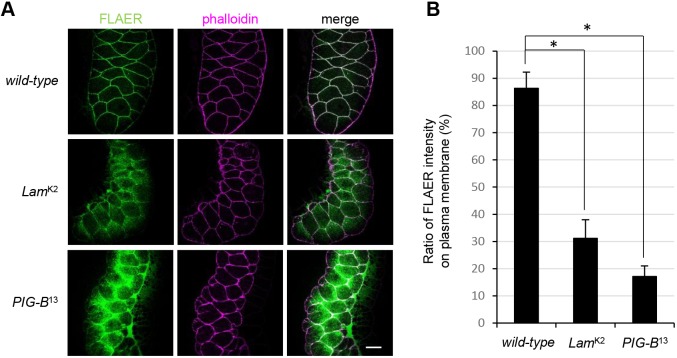
**The *Drosophila Lamin Dm0* mutant exhibits a defect in GPI-anchored proteins.** (A) Localization of GPI-anchored proteins in salivary glands of the wild-type, *Lamin Dm0* mutant (*Lam*^K2^) and *PIG-B* mutant (*PIG-B*^13^) larvae. GPI moieties were labeled with FLAER (green). The plasma membrane was labeled with rhodamine-phalloidin (magenta). Scale bar: 50 µm. (B) The ratio of FLAER signal intensity in the plasma membrane (defined by phalloidin) to that in the whole cells; salivary glands from the wild-type, *Lamin Dm0* mutant (*Lam*^K2^) and *PIG-B* mutant (*PIG-B*^13^) are shown. Data are mean±s.e.m. of data obtained in more than three specimens. *P<0.001 as determined by Student's *t*-test.

Finally, we investigated whether production of GPI-anchored proteins is impaired in a loss-of-function mutant of Lamin Dm0 (*Lam*^K2^). We detected GPI moieties in salivary glands of *Lam*^K2^, *PIG-B*^13^ and wild-type (control) larvae using fluorescently labeled inactive toxin aerolysin (FLAER), which binds to the GPI moiety. In the wild-type control, GPI-anchored proteins were expressed on the cell surface, resulting in FLAER signals along cell outlines that were rimmed by rhodamine-phalloidin (Fig. 6A). About 85% of FLAER signals were detected on the plasma membrane, as defined by phalloidin staining in the wild-type (Fig. 6B). However, the signal was also detected in the cytosol of *Lam*^K2^, similar to the pattern in *PIG-B*^13^. The ratios of the fluorescent signal intensities on the plasma membrane to those in the whole cell in the mutants were significantly reduced compared with those in the wild-type (to 31.2% in *Lam*^K2^ and 17.1% in *PIG-B*^13^). Thus, the loss-of-function mutation of Lamin Dm0 led to a defect in GPI-anchored proteins due to mislocalization of PIG-B.

## DISCUSSION

In this study, we identified Lamin Dm0 as a PIG-B-tethering protein at the INM. Although many lamin-binding proteins have been identified, this is the first report that a lamin captures an enzyme responsible for post-translational modification of plasma membrane proteins.

Lamins are involved in various biological processes. Consequently, the *Drosophila Lamin Dm0* mutant displays various tissue dysfunctions during development (e.g. smaller ovaries, underdevelopment of central nervous system ventral ganglia, a nuclear migration defect in eyes and dramatic enlargement of the ventriculus) (Osouda et al., 2005). In this study, GPI-anchored proteins were abnormally distributed in *Lam*^K2^ because of mislocalization and decreased protein expression of PIG-B. This finding suggests that some abnormalities in the *Lamin Dm0* mutant are due to incomplete GPI synthesis.

### Mechanism underlying the INM localization of PIG-B

Newly synthesized INM proteins are inserted into the ER and ONM. They then move to the INM through NPCs and are preferentially retained at the INM. Four models have been proposed to explain INM targeting: diffusion-retention, transport factor-mediated, sorting motif-mediated and vesicle-mediated (Katta et al., 2014). Diffusion-retention is the most classical model in which INM proteins diffuse from the ONM to the INM through NPCs and are preferentially retained at the INM owing to interactions with nuclear components (Holmer and Worman, 2001; Powell and Burke, 1990). To enable diffusion beyond NPCs, the size of the cytoplasmic/nucleoplasmic region of INM proteins is restricted to 60 kD (Ohba et al., 2004; Soullam and Worman, 1995). PIG-B is a multi-membrane-spanning protein and its nucleoplasmic region is only 5 kD; therefore, it can diffuse from the ER and ONM to the INM. Thereafter, PIG-B is retained at the INM by associating with a B-type lamin. However, PIG-B tended to localize to the rim of the nucleus in the peripodial membrane of *Lam*^K2^ (Fig. S2). If PIG-B diffused freely between the ER, ONM and INM, it would localize uniformly to these compartments. Therefore, PIG-B may localize to the INM via active mechanisms in addition to free diffusion. The transport factor model proposes that INM proteins contain a nuclear localization sequence (NLS) that interacts with a karyopherin in the cytoplasm (King et al., 2006; Lusk et al., 2007). This karyopherin then transports INM proteins through NPCs and releases them dependent on Ran-GTP, which is enriched inside the nucleus similar to soluble nuclear proteins. The sorting motif-mediated model suggests that INM proteins contain an INM sorting motif (Braunagel et al., 2004) that is recognized by a membrane-associated truncated version of karyopherin-α called importin-α-16 (Braunagel et al., 2007; Saksena et al., 2006). It binds to INM proteins immediately after they are translated and transfers them to the INM through the peripheral NPC. After reaching the INM, importin-α-16 releases INM proteins via a Ran-independent mechanism involving Nup50/Nup2 or other export complexes (Matsuura and Stewart, 2005). Whereas NLSs contain short stretches of basic residues, the INM sorting motif contains at least two positively charged residues within 5–8 residues that face the nucleoplasmic region and is located close to the C-terminal ends of transmembrane domains. Although a PSORT II search of PIG-B did not identify any defined NLS that has been already reported, we found one possible INM sorting motif-like stretch (^270^KKSEK^274^) in PIG-B by careful manual scanning. This sequence is located in the domains required for the NE localization of PIG-B defined in our previous study (Yamamoto-Hino et al., 2018). Moreover, replacement of residues 266–276 of PIG-B, which include the possible INM sorting motif, with the corresponding sequence of human PIG-B led to the ER localization of PIG-B. This result suggests that ^270^KKSEK^274^ functions as an INM sorting motif. Taken together, these findings indicate that PIG-B is transported by INM sorting motif-mediated machinery and is retained at the INM by Lamin Dm0.

### Lamin-binding proteins and PIG-B

The INM localization of PIG-B is dependent on its interaction with Lamin Dm0. Many lamin-interacting proteins have been reported. Here, we identified Lamin Dm0-interacting proteins in *Drosophila* S2 cells using a proximity-dependent labeling assay (Table S2). The list of highly enriched proteins includes Bicaudal D, Otefin and Lamin-B receptor, which are Lamin Dm0-binding proteins (Goldberg et al., 1998; Stuurman et al., 1999; Wagner et al., 2004). In addition, more than 50% of identified proteins were annotated as being localized in the nucleus. However, PIG-B was not detected in this experiment. This may be because the signal generated by biotinylated PIG-B was not very strong on the western blot (Fig. 3D). There are several possible reasons for this: biotinylation of PIG-B may not be effective because the nucleoplasmic domains of PIG-B are small; PIG-B peptides may be difficult to detect by mass spectrometry; or the interaction between PIG-B and Lamin Dm0 may be weak due to its indirect nature.

Some of the lamin-interacting domains have been identified. For example, emerin, a LAP2-Emerin-MAN1 (LEM)-domain protein that localizes to the INM, interacts with lamin and barrier-to-autointegration factor (BAF) via a LEM domain spanning ∼40 residues (Lee et al., 2001; Sakaki et al., 2001; Vaughan et al., 2001). Emerin, LAP2a and LAP2b (known as erbin in mammals) in vertebrates as well as Otefin and Bocksbeutel in *Drosophila* belong to this family. Another example is Sad1p, UNC-84 (SUN)-domain proteins that are tethered at the INM by their association with lamins. The lamin interaction luminal loop of SUN1 is located in the N-terminal region (Haque et al., 2010). However, the primary amino acid sequences of lamin interaction domains vary among proteins.

**Fig. 7.**
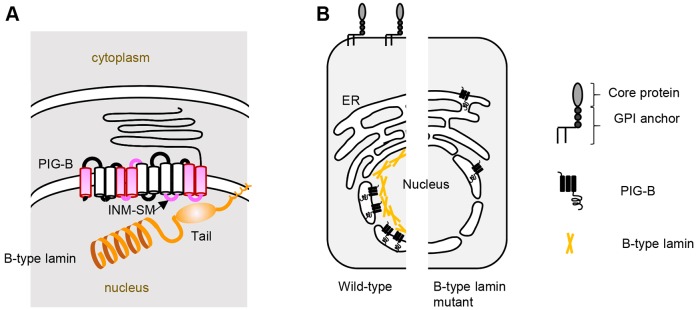
**Schematic of lamin-dependent localization of PIG-B to the INM and GPI-anchored protein synthesis.** (A) PIG-B is proximally associated with Lamin Dm0 at the INM. Magenta indicates the domains of PIG-B required for its nuclear localization. The INM sorting motif (INM-SM) is located in the fifth loop facing the nucleoplasm. The last loop, at least, directly interacts with the tail domain of Lamin Dm0. (B) PIG-B localizes to the INM via Lamin Dm0, which is required for proper synthesis of GPI-anchored proteins (left). Disruption of Lamin Dm0 results in mislocalization and degradation of PIG-B and incomplete synthesis of GPI-anchored proteins (right).

Our previous study indicates that four domains of PIG-B are required for its NE localization (Fig. 7A) (Yamamoto-Hino et al., 2018). PIG-B contains six stretches that face the nucleoplasmic side, and at least three of these are required for the NE localization of PIG-B. We showed that at least one domain (residues 319–336) is required for lamin association. Furthermore, our data suggest that PIG-B contains other sites that contribute to its interaction with Lamin Dm0. The remaining two regions that face the nucleoplasm may also associate with Lamin Dm0 and/or other proteins that facilitate the association between Lamin Dm0 and PIG-B.

We revealed that the tail domain of Lamin Dm0 is required for tethering of PIG-B at the INM. However, binding of HA-LamC_HR_D_T_ was weaker than that of HA-Lamin Dm0 in S2 cells. The head and rod domains of Lamin Dm0 may also contribute to its association with PIG-B.

### Formation of the GPI modification zone

Production of GPI-anchored proteins requires PIG-B localized to the NE. This NE localization is essential for the function of PIG-B because the ER-localizing form of PIG-B (PIG-B[ER]) does not fully rescue the lethality of the *PIG-B* mutant (Yamamoto-Hino et al., 2018). The NE and perinuclear region harbor mRNA encoding Dally-like protein (Dlp), a GPI-anchored protein that plays an important role in *Drosophila* development, and PIG-T protein, a component of the transamidase complex (TAC) that catalyzes attachment of GPI to preproteins for GPI-anchored proteins (Yamamoto-Hino et al., 2018). These results strongly suggest that the NE is part of a GPI modification zone that efficiently produces GPI-anchored proteins. In this study, we identified Lamin Dm0 as a factor that helps to form the GPI modification zone by anchoring PIG-B to the INM (Fig. 7A). In *Lam*^K2^, PIG-B was not anchored to the INM, it instead diffused to the ER and was unable to function properly (Fig. 7B). Accordingly, GPI moieties were distributed abnormally in *Lam*^K2^. Synthesis of other lipids takes place in the INM. Synthesis of phosphatidylcholine and triacylglycerol is regulated by localization of PCYT1A and Lro1 to the INM, respectively (Barbosa et al., 2019; Haider et al., 2018). The INM may provide a platform for formation of some lipids.

There are three possibilities for the biological significance of the NE localization of PIG-B, which are not mutually exclusive. First, lamin may provide a functional platform for efficient formation of GPI-anchored proteins by spatially juxtaposing the late steps of GPI synthesis by PIG-B, translation of preproteins for GPI-anchored proteins and attachment of GPI to preproteins by the TAC. Second, PIG-B may cause undesirable reactions when localized to the ER. It is unknown what products are generated by ER-localized PIG-B because most PIG-B[ER] is degraded in the ER. An *in vitro* assay of PIG-B activity must be developed. Third, PIG-B may have an unknown activity other than GPI synthesis in the nucleus. It will be interesting to investigate whether this GPI synthesis enzyme has a nuclear function at the INM, for example regulation of gene expression, chromosomal architecture and nuclear positioning.

### PIG-B stabilization and its implications for human diseases

Ten mutations of the human *PIGB* gene were recently reported to cause inherited GPI deficiencies characterized by axonal neuropathy and metabolic abnormalities (Murakami et al., 2019). Among these ten mutations, the *PIGB*^A388P^ mutant exhibits severe defects. Interestingly, this mutated amino acid (A388) is located within the region corresponding to the *Drosophila* lamin association domain, which is required for the stability of PIG-B. Accordingly, the expression level of human PIGB^A388P^ protein is very low in CHO cells and this mutant protein is unable to rescue the phenotype of PIG-B-deficient cells. These results suggest that the domains we identified are required for the stability of PIGB in human and PIG-B in *Drosophila*. Lamin Dm0 interacts with this domain in *Drosophila*, but it remains unknown which protein interacts with the corresponding domain in human. Human proteins that associate with PIGB must be identified to elucidate the molecular basis of PIGB-related diseases in the future.

## MATERIALS AND METHODS

### Fly stocks

*D. melanogaster* Canton-S (CS) was used as the wild-type strain. *PIG-B*^13^ was described previously (Yamamoto-Hino et al., 2018). *Lam*^K2^ was obtained from the Bloomington *Drosophila* Stock Center (BDSC) (stock no. 25093).

### Antibodies

The primary antibodies used in this study are listed in Table S3.

### Plasmid construction

Plasmids to express myc-tagged PIG-B proteins in S2 and CHO cells were previously described (Yamamoto-Hino et al., 2018). To construct the stable expression plasmid for PIG-B-Flag, cDNA tagged with the 3Flag epitope at the C-terminus was cloned into pMK33. cDNA encoding Lamin Dm0 was obtained from the *Drosophila* Genomic Resource Center (DGRC). cDNA encoding Lamin C was amplified from CS cDNA by RT-PCR. To construct the expression plasmid for lamins in CHO cells, cDNAs were inserted into the expression vector pCMVTNT (Promega). For the biotinylation assay, V5-miniTurboID-NES pCDNA3 (Addgene plasmid #107170) was used. A DNA fragment encoding V5-miniTurboID was ligated to the 5′ end of the PCR-amplified coding region of Lamin Dm0 cDNA and inserted into the expression vector pMK33. As a control, an expression plasmid for V5-miniTurboID was also generated. HA-tagged wild-type and chimeric lamin constructs were generated previously (Uchino et al., 2017). Each cDNA fragment was cloned into the pRmHa and pCMVTNT expression vectors.

### Cell culture and transfection

S2 cells originating from *D. melanogaster* were cultured in Schneider's medium supplemented with 10% fetal calf serum. ML-DmD9 cells originating from the wing disc of *D. melanogaster* were purchased from DGRC and cultured in M3 medium supplemented with 5% fetal calf serum, BPYE medium and 10 µg/ml insulin. These cells were transfected with DNA using calcium phosphate transfection reagent (Thermo Fisher Scientific). Stable transformants were selected using 300 µg/ml hygromycin and cloned. Protein expression was induced by incubating cells in the presence of 0.5 mM CuSO_4_ for 24 h. To knock down PIG-B-interacting proteins, double-stranded RNA for each gene and GFP (as a control) was synthesized using a T7 *In Vitro* Transcription Kit (TAKARA) and transfected into S2 and ML-DmD9 cells using calcium phosphate transfection reagent (Thermo Fisher Scientific). The primers used to generate double-stranded RNA are listed in Table S4. CHO-K1 cells (F21.3.8) were cultured in Ham's F-12 medium and transfected with plasmids using Lipofectamine 2000 (Thermo Fisher Scientific).

### Sample preparation for proteomics

S2 cells expressing PIG-B-Flag were washed with phosphate-buffered saline (PBS) and suspended in 1% formaldehyde for crosslinking. After 10 min, cells were washed three times with PBS containing 1.25 M glycine, sonicated in RIPA buffer and centrifuged. Supernatants were incubated with anti-DYKDDDDK tag antibody-conjugated magnetic beads (Wako Junyaku). After three washes with RIPA buffer, bound proteins were eluted with 0.1 M triethylamine (pH 11.5). The solutions were dried, resolved using 1% sodium deoxycholate (SDC), 2 M urea and 50 mM NH_4_HCO_3_, and incubated with trypsin at 37°C overnight. The solutions containing digested peptides were processed using phase-transfer methods to remove SDC. The obtained peptides were resolved with 0.1% formic acid and desalted using an Empore C18 disk (GL Science). The peptides were separated on a C18 reversed-phase column (75 µm×150 mm; ChromXP C18-CL 3 µm 120A; Eksigent) equipped with a nanoLC-Ultra 2D system (AB SCIEX). The masses of the eluted peptides were determined using a TripleTOF^®^ 5600 (AB SCIEX) mass spectrometer.

### Proteomics analysis by 2DICAL

This was performed as previously described (Ono et al., 2018).

### Immunoprecipitation and immunoblotting

For immunoprecipitation, cell lysates were prepared by solubilizing cells in 50 mM Tris-HCl (pH 8.0), 150 mM NaCl, 1% Triton X-100 and a protease inhibitor cocktail (Nacalai Tesque) followed by sonication. After centrifugation, supernatants were incubated with anti-Flag antibody-conjugated magnetic beads (Wako Junyaku), anti-HA antibody-conjugated magnetic beads (MBL) or an anti-lamin (ADL67.10) or rabbit anti-PIG-B antibody and Protein G Sepharose 4FastFlow (GE Healthcare). Immunoprecipitates were subjected to SDS-PAGE. The proteins were transferred to PVDF membranes (Millipore), which were blocked with PBS containing 0.05% Tween 20 and 5% skimmed milk, followed by incubation overnight with the appropriate primary antibodies. Primary antibodies were detected with horseradish peroxidase-conjugated anti-mouse, anti-rabbit and anti-rat antibodies (Jackson ImmunoResearch). Signals were visualized with Supersignal West Pico Chemiluminescent Substrate (Thermo Fisher Scientific). Images were acquired using an ImageQuant LAS 4000 mini chemiluminescence detection system (GE Healthcare).

### qPCR

Cells were frozen at −80°C and then ground up. Total RNA was extracted using an RNeasy kit (Qiagen). Superscript reverse transcriptase (Invitrogen) and oligo(dT) primers were used for reverse transcription. Real-time PCR was performed on a QuantStudio 12 K Flex system (Applied Biosystems) with Power SYBR Green (Applied Biosystems). The amount of amplified transcript was normalized against that of an internal control (rpl32). The following primers were used: Lamin Dm0 forward, 5′-ACGCCACGGTCAAGAGATAG-3′; Lamin Dm0 reverse, 5′-GATTTCCAAATCCAGGGAGA-3′; Lamin C forward, 5′-ATGCTGGTACCGCACATGAT-3′; Lamin C reverse 5′-GGAAACGTTAGCACGGACAC-3′; rpl32 forward, 5′-GCAAGCCCAAGGGTATCGA-3′; rpl32 reverse, 5′-CGATGTTGGGCATCAGATACTG-3′.

### Immunostaining

For immunofluorescence experiments, S2 cells, ML-DmD9 cells, CHO cells and tissues dissected from *Drosophila* third instar larvae were fixed in PBS containing 4% paraformaldehyde, blocked in PBS containing 0.5% bovine serum albumin and 0.1% Triton X-100, incubated with appropriate primary antibodies and then stained with Alexa Fluor 488-conjugated (Thermo Fisher Scientific) or Cy3-conjugated (Millipore) secondary antibodies and DAPI. Fluorescence images were acquired using a laser-scanning confocal microscope (LSM710, Zeiss).

### Proximity-dependent labeling assay

S2 cells harboring V5-miniTurboID-Lamin or V5-miniTurboID were incubated for 6 h in complete media supplemented with 0.5 mM CuSO_4_ and 50 µM biotin. After washing with PBS, cells were lysed in lysis buffer [50 mM Tris-HCl (pH 7.4), 150 mM NaCl, 1% SDS, 5 mM EDTA and 1 mM DTT] and sonicated. Triton X-100 was added to a final concentration of 2%. After further sonication, a 10-fold excess of RIPA buffer containing 5 mM EDTA and 1 mM DTT was added and samples were centrifuged. Supernatants were incubated with Dynabeads MyOne Streptavidin C1 (Thermo Fisher Scientific). Beads were washed three times with 2% SDS. For proteomics analysis, the beads were washed two more times with 50 mM Tris-HCl (pH 7.4), and bound proteins were trypsinized and subjected to 2DICAL analysis. For immunoblotting, bound proteins were recovered with SDS-sample buffer and heated at 95°C for 5 min.

### Statistical analysis

Data are presented as mean±s.d. of more than three independent experiments. Statistical analysis was performed using two-sided Student's *t*-tests using Microsoft Excel.

## Supplementary Material

Supplementary information

Reviewer comments
