# Supplementary material for: Lamin is essential for nuclear localization of the GPI synthesis enzyme PIG-B and GPI-anchored protein production in Drosophila
Source: J Cell Sci. 2020 Mar 26;133(6):jcs238527. doi: 10.1242/jcs.238527 (PMC7104860; doi:10.1242/jcs.238527)
Supplement: Supplementary information [file joces-133-238527-s1.pdf]

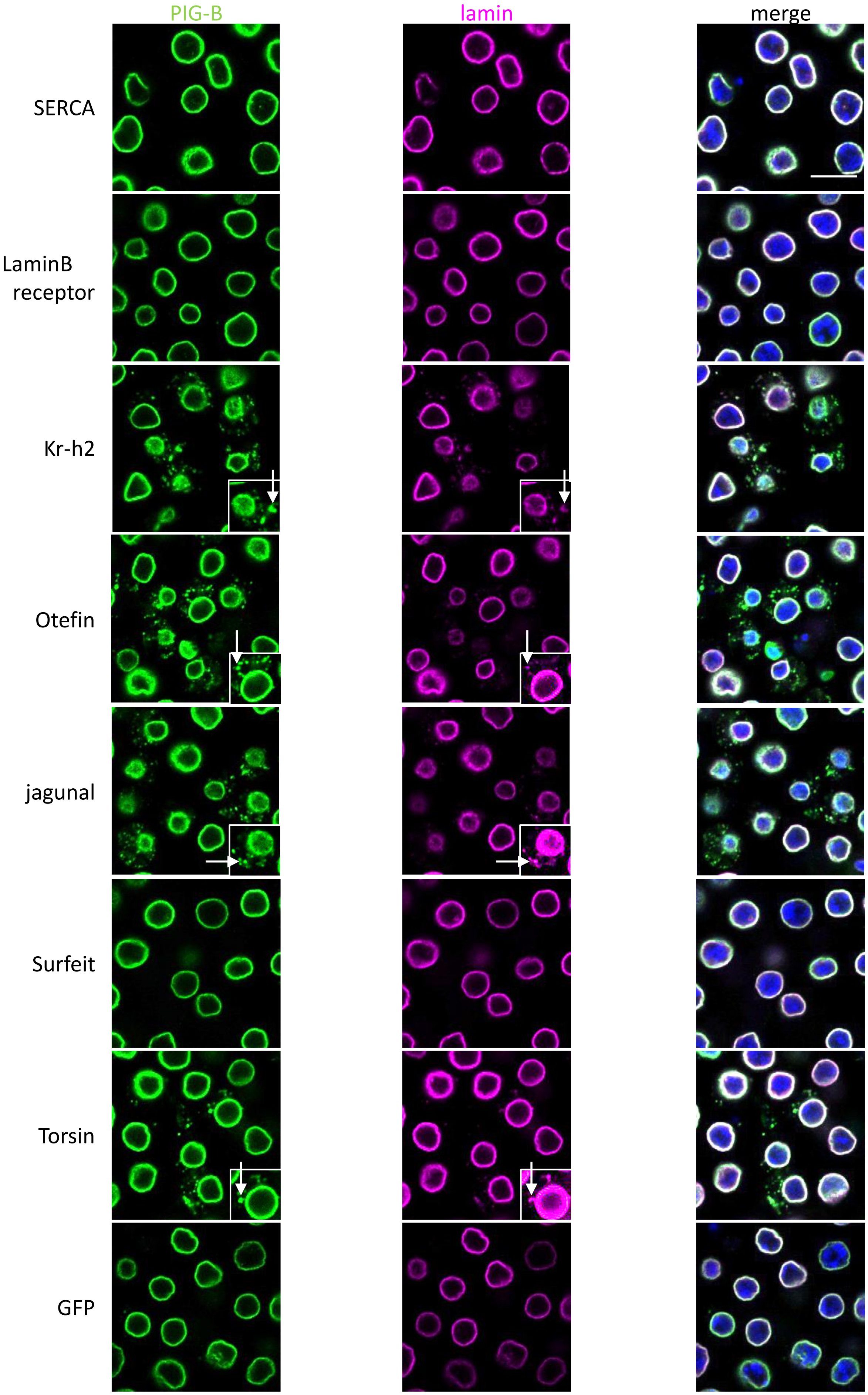

Figure. S1 Localization of PIG-B in S2 cells depleted of the PIG-B-binding molecules shown in Table 1

Cells were stained with an anti-PIG-B antibody (green), an anti-Lamin Dm0 antibody (magenta), and DAPI (blue). The insets show enlarged images. White arrows indicate PIG-B localized to the cytoplasm with Lamin Dm0. Bar, 10 μm.

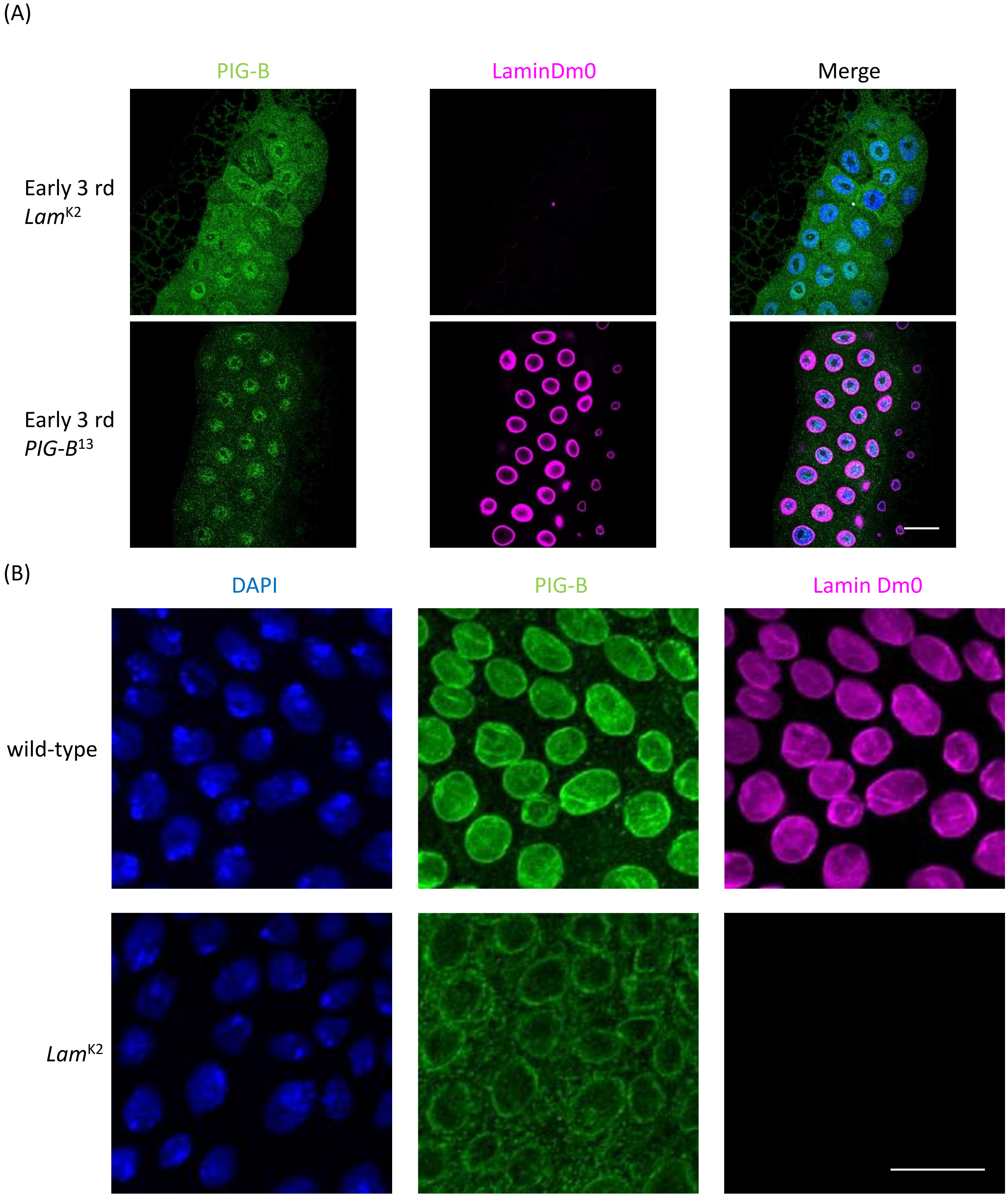

Figure S2 Localization of PIG-B in the *Lamin Dm0* and *PIG-B* mutants.
(A) Immunofluorescence analysis of PIG-B in salivary glands of early third instar larvae of the *Lamin Dm0* mutant (*Lam^{K2}*) and the *PIG-B* mutant (*PIG-B^{13}*). Salivary glands were stained with an anti-PIG-B antibody (green), an anti-Lamin Dm0 antibody (magenta), and DAPI (blue). Bar, 40 μm.
(B) Immunofluorescence analysis of PIG-B in the peripodial membrane of the wild-type, *Lamin Dm0* mutant (*Lam^{K2}*). The peripodial membrane were stained with an anti-PIG-B antibody (green), an anti-Lamin Dm0 antibody (magenta), and DAPI (blue). Bar, 10 μm.

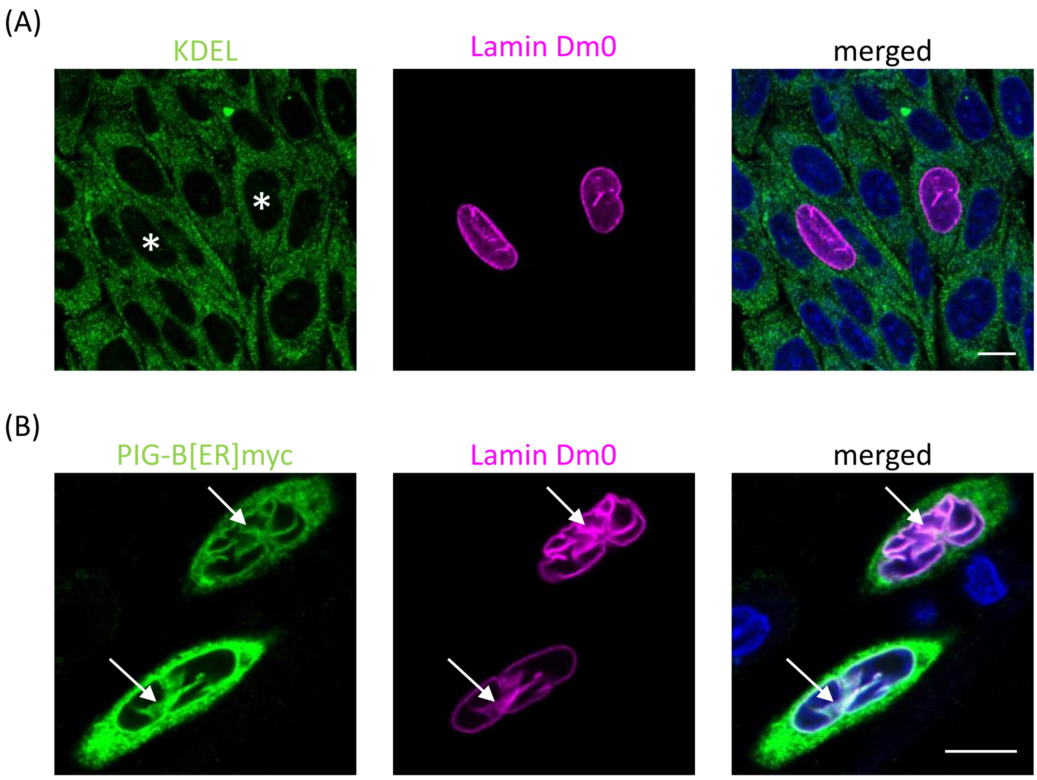

Figure S3 Immunostaining of Lamin Dm0-expressing CHO cells.

(A) Distribution of ER in CHO cells. CHO cells were stained with an anti-KDEL antibody (ER marker) (green), an anti-Lamin Dm0 antibody (magenta), and DAPI (blue). Asterisks indicate CHO cells expressing Lamin Dm0. No difference of appearance of the ER was observed between Lamin Dm0 expressing and no expressing cells. Bar, 10 μm.

(B) Immunofluorescence analysis of CHO cells expressing ER-localized myc-tagged PIG-B (PIG-B[ER]myc) together with Lamin Dm0 strongly. Cells were stained with an anti-myc antibody (green), an anti-Lamin Dm0 antibody (magenta), and DAPI (blue). White arrows indicate co-localization of PIG-B[ER]myc and Lamin Dm0 in the nucleoplasm. Bar, 10 μm.

Development • Supplementary information

Table S1. 2DICAL data for PIG-B-associated proteins obtained from immunoprecipitation experiments

Proteins identified by immunoprecipitation experiments followed by 2DCAL are listed in order of fold changes between precipitates with PIG-B and the control. (column A) Protein ID. (column B) Protein description and gene name (GN). (column C) Average signal intensities of identified peptides from immunoprecipitates in S2 cells. (column D,E) Average signal intensities of identified peptides in each experiment from immunoprecipitates in PIG-B-Flag expressing S2 cells. (column F) ave (PIG-B-Flag IP 1,2) normalized by control. (column G) Subcellular localization annotated by Uniprot. Red character indicates selected genes in Table 1.

Click here to Download Table S1

Table S2. 2DICAL data for lamin-associated proteins obtained from proximity-dependent labeling

Proteins identified by a proximity-dependent labeling assay with Lamin Dm0-TuboID followed by 2DCAL are listed in order of fold changes between precipitates labelled with Lamin Dm0-TuboID and the control. (column A) Protein ID. (column B) Protein description and gene name (GN). (column C,D,E) Average signal intensities of identified peptides in each experiment from V5-mT-Lamin biotinylated fraction. (column F, G, H) Average signal intensities of identified peptides in each experiment from V5-mT biotinylated fraction. (column I) Average of signal intensity from V5-mT-Lamin (C, D, E). (column J) Average of signal intensity of V5-mT (F, G, H). (column K) V5-mT-Lamin normalized by V5-mT (I/J). (column L) p-value (C,D,E vs. F,G,H). Statistical analysis was performed with Student's t-tests using Microsoft Excel. (column L) Subcellular localization annotated by Uniprot.

Click here to Download Table S2

Development • Supplementary information

Table S3 Primary antibodies used in this study

antibody	Description	Cat. No.	lot No.	dilution	
				Immuno blot	Immuno staining
rabbit anti-PIG-B	Yamamoto-Hino et. al., (2018)			1:20000	1:10000
mouse anti-DYKDDDDK tag 1E6	Wako Junyaku	012-22383	WDL0641	1:20000	-
rabbit anti-myc A14	Santa cruz	sc-789	H1308	-	1:500
rabbit anti-V5 D3H8Q	Cell signaling	#13202		1:2000	-
mouse anti-V5	Invitrogen	R960-25	1869182	-	1:1000
rat anti-HA 3F10	Roche	#11867423001		1:2000	1:500
mouse anti-lamin Dm0 ADL67.10	Developmental Studies Hybridoma Bank (DSHB)			1:2000	1:1000
mouse anti-lamin C LC28.20	Developmental Studies Hybridoma Bank (DSHB)			-	1:500
rat anti-αtubulin	Oxford Biotechonology	OBT0045	1002	1:2000	-
mouse anti-KDEL 10C3	Stressgen	SPA-827	B404464	-	1:500
Streptavidin-HRP	abcam	ab7403	GR3230032-5	1:10000	-
Alexa Fluor 488–labeled Streptavidin	Thermo	s11223		-	1:500
Alexa Fluor 488–labeled FLAER	CEDARLANE	FL1S-C	1803/3A	-	1 mM
Rhodamin-labeled phalloidin	Thermo	R415	2105247	-	1:250

Development • Supplementary information

Table S4 Primers used to generate double-stranded RNA in this study

CG No.	Name	sequence (5'-3')
CG3725	3725R F1	CCATTGAGGCCCTCAAGGAGTACG
	3725R R1	GTCGTTGAAGTGGCCGATGTTGATG
	T73725R F1	gatcactaatacgactcactatagggCCATTGAGGCCCTCAAGGAGTACG
	T73725R R1	gatcactaatacgactcactatagggGTCGTTGAAGTGGCCGATGTTGATG
CG6944	Lam 1306-1806 dsRNA F1	CCATCGCGTCGCACTCCCTC
	Lam 1306-1806 dsRNA R1	TTGCCGTCCACGGCGGTCAC
	T7Lam 1306-1806 dsRNA F1	gatcactaatacgactcactatagggCCATCGCGTCGCACTCCCTC
	T7Lam 1306-1806 dsRNA R1	gatcactaatacgactcactatagggTTGCCGTCCACGGCGGTCAC
CG6202	6202R F1	CGAGTACATAGCGAAAACGGAGG
	6202R R1	TGATGAACATGAAGGCCAGCAGG
	T76202R F1	gatcactaatacgactcactatagggCGAGTACATAGCGAAAACGGAGG
	T76202R R1	gatcactaatacgactcactatagggTGATGAACATGAAGGCCAGCAGG
CG3024	TorsinIR F1	ATGATGAGCTTTCCACGCATGTTA
	TorsinIR R1	CTACGCAAGGTATCGCGCACCGC
	T7TorsinIR F1	gatcactaatacgactcactatagggATGATGAGCTTTCCACGCATGTTA
	T7TorsinIR R1	gatcactaatacgactcactatagggCTACGCAAGGTATCGCGCACCGC
CG17952	GD2133 F1	CGCAAACGCATAACCGAACGACTG
	GD2133 R1	TGGGCAGAAGTAGCAGGAACAGG
	T7GD2133 F1	gatcactaatacgactcactatagggCGCAAACGCATAACCGAACGACTG
	T7GD2133 R1	gatcactaatacgactcactatagggTGGGCAGAAGTAGCAGGAACAGG
CG10978	10978R F1	GCACCGATGGCAACGACTTCGAG
	10978R R1	CGTGCACCTGGAAGCCCACAAAG
	T710978R F1	gatcactaatacgactcactatagggGCACCGATGGCAACGACTTCGAG
	T710978R R1	gatcactaatacgactcactatagggCGTGCACCTGGAAGCCCACAAAG
CG9159	9159R 1F	AACCGCAGCAATCTCAATCACAG
	9159R 2R	AGGCATGATGAAGATCTCGCAGA
	T79159R 1F	gatcactaatacgactcactatagggAACCGCAGCAATCTCAATCACAG
	T79159R 2R	gatcactaatacgactcactatagggAGGCATGATGAAGATCTCGCAGA
CG5581	5581R F1	ATGCTGGCACAGGGGCTGCCCAAC
	5581R R1	CTCCTCTCGATCAGCGCGCTTCG
	T75581R F1	gatcactaatacgactcactatagggATGCTGGCACAGGGGCTGCCCAAC
	T75581R R1	gatcactaatacgactcactatagggCTCCTCTCGATCAGCGCGCTTCG
CG10119	LamC 1269-1768 dsRNA F1	GACCGCCAGTGCCAGTTCCC
	LamC 1269-1768 dsRNA R1	AGGTGTGGCTGGAAACGTTA
	T7LamC 1269-1768 dsRNA F1	gatcactaatacgactcactatagggGACCGCCAGTGCCAGTTCCC
	T7LamC 1269-1768 dsRNA R1	gatcactaatacgactcactatagggAGGTGTGGCTGGAAACGTTA

Development • Supplementary information
