# Supplementary material for: Lamin is essential for nuclear localization of the GPI synthesis enzyme PIG-B and GPI-anchored protein production in Drosophila
Source: J Cell Sci. 2020 Mar 26;133(6):jcs238527. doi: 10.1242/jcs.238527 (PMC7104860; doi:10.1242/jcs.238527)
Supplement: Reviewer comments [file joces-133-238527_review_history.pdf]

Journal of Cell Science

Lamin is essential for nuclear localization of the GPI synthesis enzyme PIG-B and GPI-anchored protein production in *Drosophila*

Miki Yamamoto-Hino, Kohei Kawaguchi, Masaya Ono, Kazuhiro Furukawa and Satoshi Goto
DOI: 10.1242/jcs.238527

Editor: David Stephens

Review timeline
Original submission: 25 August 2019
Editorial decision: 23 September 2019
First revision received: 24 January 2020
Accepted: 28 January 2020

Original submission

First decision letter

MS ID#: JOCES/2019/238527

MS TITLE: Lamin is essential for localization of the GPI synthesis enzyme PIG-B to the inner nuclear membrane and production of GPI-anchored proteins

AUTHORS: Miki Yamamoto-Hino, Kohei Kawaguchi, Masaya Ono, Kazuhiro Furukawa, and Satoshi Goto
ARTICLE TYPE: Research Article

We have now reached a decision on the above manuscript.

To see the reviewers' reports and a copy of this decision letter, please go to: https://submit-jcs.biologists.org and click on the 'Manuscripts with Decisions' queue in the Author Area. (Corresponding author only has access to reviews.)

As you will see, the reviewers raise a number of substantial criticisms that prevent me from accepting the paper at this stage. They suggest, however, that a revised version might prove acceptable, if you can address their concerns. If you think that you can deal satisfactorily with the criticisms on revision, I would be pleased to see a revised manuscript. We would then return it to the reviewers.

Most of the comments can be addressed through revisions to the text. Some conclusions should be toned down unless further support is given from experimental data (such as a direct protein interaction). The proteomics data require some additional comment. It would be ideal if you could determine whether the lamin B mutation affects surface presentation of GPI-APs.

Please ensure that you clearly highlight all changes made in the revised manuscript. Please avoid using 'Tracked changes' in Word files as these are lost in PDF conversion.

I should be grateful if you would also provide a point-by-point response detailing how you have dealt with the points raised by the reviewers in the 'Response to Reviewers' box. Please attend to all of the reviewers' comments. If you do not agree with any of their criticisms or suggestions please explain clearly why this is so.

© 2020. Published by The Company of Biologists under the terms of the Creative Commons Attribution License (https://creativecommons.org/licenses/by/4.0/).

Reviewer 1

Advance summary and potential significance to field

Goto et al report an interesting finding that in Drosophila, one of the enzymes in the GPI-AP synthesis pathway, PIG-B, localizes to a subdomain of the nuclear envelope via interaction with a B-type lamin Dm0, which may be important for proper cell-surface expression of GPI-APs. Tail domain of Dm0 is required for the interaction. In contrast, lamin C does not affect DPIG-B localization. This finding builds on their previous work (Yamamoto-Hino, JCS 2018), where they showed that Dros PIG-B localizes to the NE, as well as that this localization affects cell-surface expression of GPI-APs

This work is overall interesting and relevant, given the importance and abundance of GPI-APs. In addition, the findings of Goto et al may be related to other recent work that has demonstrated the localization of lipid biosynthesis enzymes to the inner nuclear membrane in different organisms (for example, Haider, Dev Cell 2018, Barbosa, Dev Cell 2019; the authors currently limit their discussion to GPI-APs, but may want to consider these other examples).

Comments for the author

In general, the experiments can be presented and organized better, it is not always clear what is new and what has been presented before (specifically in Yamamoto-Hino, 2018), and the authors should be more careful to not overstate their conclusions. Quantification of some of the presented experiments would also be useful.

Specific comments:

1. The word ‘Drosophila’ should be added to the title, as there is no evidence that PIG-B associates with nuclear envelope in other species.

2. Proteomic analysis of DPIG-Flag binding partners:
The hits are presented in Table S1. The authors state that they further analyzed hits with >2x higher factor of enrichment that localize to the NE and/or the ER, which are listed in Table 1, lamin Dm0 being one of these hits. However, by a quick look, I could find a number of other ER proteins among the significant hits in Table S1 (SAC1, FIT1, calreticulin, Derlin, ERD2); therefore, Table 1 is misleading, and the author should clarify how they selected the candidates that they analyzed further. It would be good to add an overview of the hits in Table S1 (by category or localization). Also, Table S1 could be presented better and it should be explained what is shown in different columns.

3. I am not entirely convinced that DPIG and Lam Dm0 interact directly – this in not directly shown, but is stated very strongly in several places. More careful wording would be appropriate.

The strongest evidence for direct interaction comes from the heterologous expression of the two proteins in CHO cells, where Lam Dm0 expression is sufficient to bring DPIG to the NE. But there are some discrepancies:
- In Fig. 5, the chimera LamC(HR)D(T) rescues the localization of DPIG-B better than full-length Lam Dm0, but by Co-IP this chimera brings down much less DPIG-B than full-length Lam Dm0.
- What are the sites of interaction in the two proteins? If PIG-B requires multiple regions to localize to NE, this may also suggest that other proteins are involved in the interaction or in PIG-B. Discussion of the contribution of the of the 339loop requires some quantification of localization data; how big is the difference in localization of DPIG-B[ER] in control vs. Lamin-expressing cells in 2E? (for one condition, a single cell with an arrow pointing to a small detail is shown). Lamin expression could also change the appearance of the ER, so an ER marker should also be shown here.

4. The experiment with Lam Dm0-TurboID fusion could address the above point, except that it is not shown what partners of Lam Dm0 are identified by this approach. DPIG-B among the biotinylated proteins can be detected by Western blot (the signal is not very strong); it is not clear if this result is significant. Can DPIG-B be identified by proteomics? Which other proteins are biotinylated? (gel in Fig. 3A and images in 3B would suggest that many are). As is, I don’t see why

© 2020. Published by The Company of Biologists under the terms of the Creative Commons Attribution License (https://creativecommons.org/licenses/by/4.0/).

this experiment is shown; it is not clear whether the TurboID approach is working, and the authors already show in Fig 2A that lamin and DPIG-B co-IP.

5. Does lamin B mutation affect surface presentation of GPI-APs, as the authors have already shown for DPIG-B in Yamamoto-Hino 2018? (Fig 6) How similar are the phenotypes of mutants in the two genes?
- The explanation of this figure needs to be improved: What are the mutant flies used (please add reference for text in this figure, state that these are null alleles); some wording is confusing (the proteins are not depleted therefore the proteins are not expressed, not 'depleted'); at what stage are the cells from these flies imaged? -- I understood that PIG-B13 mutant flies were embryonic lethal. What is the general phenotype of LamK2 mutant flies?
- It is difficult to say to what extent FLAER staining is similar in PIG-B13 and in LamK2 cells, although it is clear that both mutants look different than wild-type. (Phalloidin staining also looks different in the two mutants). It would be useful to also follow a specific GPI-AP, like Dlp (as in Yamamoto-Hino). Could levels of Dlp in the cells of different flies be checked by Western blot?

6. Introduction:
Line 56-59: Ref Castillon, Traffic 2009; 10: 186–200 could be added here; this work suggests that GPI-APs and non-GPI proteins are sorted into different ER exit sites, so it seems more directly relevant than Muniz 2001. It would be fair to be more specific in quoting this work: it is not clear at this point whether or how the sorting of mature GPI-APs to a specific ERES is related to localization of PIG-B to NE, so I find the current sentence a bit misleading. Similarly, Vidugiriene, 1999, reported that de-N-acetylation of GlcNAc-PI, but not other enzymatic activities in the GPI-AP pathway, was confined to a mitochondria-associated ER subcompartment.

On the other hand, some lipid modifying enzymes have recently been observed localizing to the inner nuclear membrane, see Haider 2018 Developmental Cell 45, 481–495 for PCY1A or Barbosa 2019 Developmental Cell 50, 1–12 for Lro1 in yeast, which associates with a nuclear membrane subdomain, similarly to PIG-B.

Minor comments:
- Abstract: I suggest replacing "two-dimensional image-converted analysis of liquid chromatography and mass spectrometry" with "proteomic analysis"

- In Fig. 5, HA-tagged Lamin Dm0 and Lamin C have a different name than in other figures – this is confusing, please use the same name. The hatched pattern for Lamin B in 5A is also confusing; simple grey fill would be better.

- Some abbreviations seem unnecessary and complicate the reading:
INM-SM: spell out 'sorting motif'
What is LEM family?
What is SUN family?

- Lines 271-272: Replace 'domain' with 'luminal loop'

<u>Reviewer 2</u>

Advance summary and potential significance to field

The manuscript by Yamamoto-Hino reports that nuclear lamin B in Drosophila (lamin Dm0) is required for localisation of phosphatidylinositol glycan (PIG-B), an enzyme involved in the synthesis of glycosylphosphatidylinositol (GPI). This represents an important advance in the characterisation of GPI-related processes and a novel role to nuclear lamins. In a previous study, the authors found that failure of PIG-B to localise to the nuclear envelope (NE) correlates with lethality in Drosophila. To understand the mechanism by which PIG-B is recruited to the NE, the authors purified DIG-B-interacting proteins from S2 cells, ending up with a list of ~100 enriched proteins. Among these are Dm0 and lamin B receptor. The interaction of PIG-B with Dm0 is confirmed by reciprocal co-IP of endogenous proteins and by biotin proximity labelling. Depletion of Dm0 by RNAi or mutation prevents PIG-B NE accumulation. Conversely, by ectopic co-expression of Dm0 and PIG-B in CHO

© 2020. Published by The Company of Biologists under the terms of the Creative Commons Attribution License (https://creativecommons.org/licenses/by/4.0/).

cells, the former is able to recruit the latter to the NE. The interaction with Dm0 is specific in the sense that lamin C is not required for PIG-B localisation. Chimeras between lamins Dm0 and C demonstrates that the C-terminus of Dm0 is essential but not fully sufficient to recruit PIG-B. Importantly, the cellular distribution of GPI-anchored proteins is severely and similarly affected in Dm0 and PIG-B mutants.

Comments for the author

The manuscript is very well-written, clear and attractive. The quality of the experimental data seems very good and the authors discuss carefully their results in relation to the current status of the field. They propose several models for how PIG-B accumulates in the nucleus and why this is important. While the current data can stand on their own, I find it would strengthen that manuscript considerably if at least some of these possibilities were experimentally addressed. In particular, to determine if the interaction between Dm0 and PIG-B is required ‘only’ to position PIG-B at the NE or if Dm0 has an additional role in PIG-B activity, the authors could fuse PIG-B to an inner nuclear membrane protein (e.g. otefin) and test for rescue of GPI defects in Dm0 mutants.

Specific points:

The data in Table S1 suggest that only a single control sample was used in the proteomics study. Please comment.

I agree that the data presented are compatible with, and in favour of a model in which Dm0 and PIG-B interact directly and physically with each other, but definite evidence is lacking. All the interaction assays were performed in complex cellular contexts, thus, it cannot be ruled out that the interaction is indirect. I suggest this is commented in the manuscript.

In Figure 5 (and in the text), LamC is written as LamCHRCT. It would be simpler to maintain the name as LamC and only use the HR/T subscripts for the two chimeras.

Phalloidin staining in the LamK2 mutant is more intense than in control flies, whereas the signal is weaker in the PIB-B13 mutant. Is this consistent and other samples and, if so, what could be the explanation?

First revision

<u>Author response to reviewers' comments</u>

Most of the comments can be addressed through revisions to the text. Some conclusions should be toned down unless further support is given from experimental data (such as a direct protein interaction). The proteomics data require some additional comment. It would be ideal if you could determine whether the lamin B mutation affects surface presentation of GPI-APs.

RE: Thank you for your helpful suggestions. We have toned down our conclusions as requested. We have also included comments concerning the proteomics data (see the footnotes in the supplemental data file and response to Reviewer #2 in this letter). To show decreased surface presentation of GPI-APs, we calculated the ratio of the GPI-AP signal between the cell surface and the intracellular space. Please refer to our response to comment #5 by Reviewer #1.

We have a strict limit for the title of the paper to be under 120 characters, including spaces. As your title length (139 characters) currently exceeds this, we would appreciate it if you could reduce the length of your title before you re-upload your files.

RE: We have reduced the character count of the title to 113 (including spaces).

Reviewer 1 Advance summary and potential significance to field

© 2020. Published by The Company of Biologists under the terms of the Creative Commons Attribution License (https://creativecommons.org/licenses/by/4.0/).

Goto et al report an interesting finding that in Drosophila, one of the enzymes in the GPI-AP synthesis pathway, PIG-B, localizes to a subdomain of the nuclear envelope via interaction with a B-type lamin Dm0, which may be important for proper cell-surface expression of GPI-APs. Tail domain of Dm0 is required for the interaction. In contrast, lamin C does not affect DPIG-B localization. This finding builds on their previous work (Yamamoto-Hino, JCS 2018), where they showed that Dros PIG-B localizes to the NE, as well as that this localization affects cell-surface expression of GPI-APs

This work is overall interesting and relevant, given the importance and abundance of GPI-APs. In addition, the findings of Goto et al may be related to other recent work that has demonstrated the localization of lipid biosynthesis enzymes to the inner nuclear membrane in different organisms (for example, Haider, Dev Cell 2018, Barbosa, Dev Cell 2019; the authors currently limit their discussion to GPI-APs, but may want to consider these other examples).

Reviewer 1 Comments for the author
In general, the experiments can be presented and organized better, it is not always clear what is new and what has been presented before (specifically in Yamamoto-Hino, 2018), and the authors should be more careful to not overstate their conclusions. Quantification of some of the presented experiments would also be useful.

Specific comments:

1. The word 'Drosophila' should be added to the title, as there is no evidence that PIG-B associates with nuclear envelope in other species.

RE: As the reviewer suggests, the words "in Drosophila" have been added to the title.

2. Proteomic analysis of DPIG-Flag binding partners:
The hits are presented in Table S1. The authors state that they further analyzed hits with >2x higher factor of enrichment that localize to the NE and/or the ER, which are listed in Table 1, lamin Dm0 being one of these hits. However, by a quick look, I could find a number of other ER proteins among the significant hits in Table S1 (SAC1, FIT1, calreticulin, Derlin, ERD2); therefore, Table 1 is misleading, and the author should clarify how they selected the candidates that they analyzed further. It would be good to add an overview of the hits in Table S1 (by category or localization). Also, Table S1 could be presented better and it should be explained what is shown in different columns.

RE: I agree that table S1 was misleading. We first selected Lamin Dm0, Torsin, Lamin-B receptor, Kr-h2, and Otefin, all of which localize to the NE. Then, we selected SERCA, Surfeit 4, and Jagunal, all of which interact with the five selected proteins (Table 1). We have revised the text accordingly (lines 109–111). In addition, we have added a "subcellular localization" column to Table S1 and provided an explanation of each column in a footnote.

3. I am not entirely convinced that DPIG and Lam Dm0 interact directly – this in not directly shown, but is stated very strongly in several places. More careful wording would be appropriate.

RE: I agree that our data do not demonstrate a convincing direct interaction between DPIG-B and Lamin Dm0. It is difficult to show a direct interaction using purified DPIG-B and Lamin Dm0 because E. coli expressed very little DPIG-B. Therefore, we have revised the manuscript text such that the wording is more careful.

The strongest evidence for direct interaction comes from the heterologous expression of the two proteins in CHO cells, where Lam Dm0 expression is sufficient to bring DPIG to the NE. But there are some discrepancies:
- In Fig. 5, the chimera LamC(HR)D(T) rescues the localization of DPIG-B better than full-length Lam Dm0, but by Co-IP this chimera brings down much less DPIG-B than full-length Lam Dm0.

RE: Although we cannot provide a precise answer as to why LamC(HR)D(T) pulled down much less DPIG-B than full-length Lam Dm0 in the Co-IP experiments (Fig. 5C), we think that the interaction between LamC(HR)D(T) and PIG-B may be weaker than that between full-length Lam Dm0 and PIG-

© 2020. Published by The Company of Biologists under the terms of the Creative Commons Attribution License (https://creativecommons.org/licenses/by/4.0/).

B. The Co-IP experiment involved several washing steps; this may have removed a large amount of PIG-B, which binds more weakly to LamC(HR)D(T) than to full-length Lam Dm0. By contrast, washing during cell staining is less harsh than that during Co-IP, resulting in much more residual PIG-B (Fig. 5D). Thus, NE localization of DPIG-B with LamC(HR)D(T) appears comparable with localization with full-length Lam Dm0.

- What are the sites of interaction in the two proteins? If PIG-B requires multiple regions to localize to NE, this may also suggest that other proteins are involved in the interaction or in PIG-B. Discussion of the contribution of the of the 339loop requires some quantification of localization data; how big is the difference in localization of DPIG-B[ER] in control vs. Lamin-expressing cells in 2E? (for one condition, a single cell with an arrow pointing to a small detail is shown). Lamin expression could also change the appearance of the ER, so an ER marker should also be shown here.

RE: I agree with your suggestion that there may be other proteins involved in the interaction between PIG-B and Lamin Dm0 because multiple regions of PIG-B are required for the interaction. The contribution of the loop (319–336) can be described as follows. We suggest that a portion of DPIG-B[ER] co-localized with Lamin Dm0 in the nucleoplasm (arrows, Fig. 2E, lower). This co-localization was more apparent in CHO cells that strongly express Lamin Dm0 (Fig. S3B). Approximately 95% of cells expressing both Lamin Dm0 and DPIG-B[ER] showed co-localization in the nucleoplasm (25 of 26 cells), while only 17% of cells expressing only DPIG-B[ER] showed nucleoplasmic localization of DPIG-B[ER] (4 out of 23 cells). These results suggest that regions other than the loop (319–336) play a role in association with Lamin Dm0, even if it is unclear whether the interaction is direct or indirect. These results are presented in lines 160–164 of the revised manuscript.
Regarding the Reviewer's second concern, we addressed this by immunostaining the ER with an anti-KDEL antibody (Fig. S3A). The result clearly shows that Lamin expression did not alter ER morphology (see lines 150–153 of the revised manuscript).

4. The experiment with Lam Dm0-TurboID fusion could address the above point, except that it is not shown what partners of Lam Dm0 are identified by this approach. DPIG-B among the biotinylated proteins can be detected by Western blot (the signal is not very strong); it is not clear if this result is significant. Can DPIG-B be identified by proteomics? Which other proteins are biotinylated? (gel in Fig. 3A and images in 3B would suggest that many are). As is, I don't see why this experiment is shown; it is not clear whether the TurboID approach is working, and the authors already show in Fig 2A that lamin and DPIG-B co-IP.

RE: The experiment with the Lam Dm0-TurboID fusion was performed to examine whether DPIG-B faces the nucleoplasm. The finding that DPIG-B was biotinylated by Lam Dm0-TurboID suggests that DPIG-B faces the nucleoplasm. To test whether this experiment works, we performed an additional experiment with Lam Dm0-TurboID and identified Lamin Dm0-interacting proteins by proteomics analysis. We detected several proteins that either localize at the inner nucleus or interact with Lam Dm0 (table S2). Examples include Bicaudal-D, Otefin, and Lamin B receptor. Thus, we conclude that the experiment with Lam Dm0-TurboID identified proteins positioned proximally to Lam Dm0. This result is presented in lines 179–188 and in table S2. However, we did not detect DPIG-B in this experiment. This may be because the signal generated by biotinylated PIG-B was not very strong on the Western blot (Fig. 3D). There are several possible reasons for this: biotinylation of DPIG-B may be ineffective because the nucleoplasmic domains of DPIG-B are small; DPIG-B peptides may be difficult to detect by mass spectrometry; or interaction between DPIG-B and Lamin Dm0 may be weak due to an indirect interaction. These possibilities are mentioned in the revised manuscript (lines 287–294).

5. Does lamin B mutation affect surface presentation of GPI-APs, as the authors have already shown for DPIG-B in Yamamoto-Hino 2018? (Fig 6) How similar are the phenotypes of mutants in the two genes?

RE: We quantified the phenotypes of the wild type, LamK2, and PIG-B13 mutants by measuring the relative intensity of FLAER signals at the plasma membrane and in the whole cell (Fig. 6B). The results clearly show that defects are comparable between LamK2 and PIG-B13 mutants, indicating that Lamin Dm0 is required for surface presentation of GPI-APs. This result is described in lines 231–237 and Fig. 6B in the revised manuscript.

© 2020. Published by The Company of Biologists under the terms of the Creative Commons Attribution License (https://creativecommons.org/licenses/by/4.0/).

- The explanation of this figure needs to be improved: What are the mutant flies used (please add reference for text in this figure, state that these are null alleles); some wording is confusing (the proteins are not depleted therefore the proteins are not expressed, not 'depleted'); at what stage are the cells from these flies imaged? -- I understood that PIG-B13 mutant flies were embryonic lethal. What is the general phenotype of LamK2 mutant flies?

RE: Thank you for the comments. The alleles LamK2 and PIG-B13 used in this figure are described for the first time in lines 119–121 and 124–125. Since these alleles are larval lethal, we analyzed salivary glands at the larval stage. We have included this information in the text and figure legend. In addition, we have corrected the statement (removal of 'depleted') in lines 227–228.

- It is difficult to say to what extent FLAER staining is similar in PIG-B13 and in LamK2 cells, although it is clear that both mutants look different than wild-type. (Phalloidin staining also looks different in the two mutants). It would be useful to also follow a specific GPI-AP, like Dlp (as in Yamamoto-Hino). Could levels of Dlp in the cells of different flies be checked by Western blot?

RE: As described above, we quantified the phenotypes and found that the LamK2 and PIG-B13 mutants have comparable defects. In the present study, we used mainly salivary glands for in vivo studies; however, Dlp is not expressed in this tissue. Thus, we are not able to examine levels of Dlp.

6. Introduction:
Line 56-59: Ref Castillon, Traffic 2009; 10: 186–200 could be added here; this work suggests that GPI-APs and non-GPI proteins are sorted into different ER exit sites, so it seems more directly relevant than Muniz 2001. It would be fair to be more specific in quoting this work: it is not clear at this point whether or how the sorting of mature GPI-APs to a specific ERES is related to localization of PIG-B to NE, so I find the current sentence a bit misleading. Similarly, Vidugiriene, 1999, reported that de-N-acetylation of GlcNAc-PI, but not other enzymatic activities in the GPI-AP pathway, was confined to a mitochondria-associated ER subcompartment.

RE: Thank you for the suggestion. I have revised the introduction as suggested (lines 55–59).

On the other hand, some lipid modifying enzymes have recently been observed localizing to the inner nuclear membrane, see Haider 2018 Developmental Cell 45, 481–495 for PCY1A or Barbosa 2019 Developmental Cell 50, 1–12 for Lro1 in yeast, which associates with a nuclear membrane subdomain, similarly to PIG-B.

RE: Thank you for your helpful suggestion. I have referred to these papers in the Discussion section of the revised manuscript (lines 326–329).

Minor comments:
- Abstract: I suggest replacing "two-dimensional image-converted analysis of liquid chromatography and mass spectrometry" with "proteomic analysis"

RE: I have revised the text as suggested (line 35).

- In Fig. 5, HA-tagged Lamin Dm0 and Lamin C have a different name than in other figures – this is confusing, please use the same name. The hatched pattern for Lamin B in 5A is also confusing; simple grey fill would be better.

RE: I have changed "Lamin C(HR)C(T)" to "Lamin C" and used gray fills in Figure 5A (instead of the hatched pattern).

- Some abbreviations seem unnecessary and complicate the reading:
INM-SM: spell out 'sorting motif'

RE: I changed "INM-SM" to "INM sorting motif" in the revised manuscript (lines 267–281).

What is LEM family?

© 2020. Published by The Company of Biologists under the terms of the Creative Commons Attribution License (https://creativecommons.org/licenses/by/4.0/).

RE: The term “LEM family” may be inaccurate. Thus, I have changed “LEM family” to “LEM (LAP2-Emerin-MAN1) domain proteins” (lines 295–296). As you know, LEM (LAP2-Emerin-MAN1) domain proteins interact with Lamin, DNA binding proteins, and DNA. Thus, they localize to the INM.

What is SUN family?

RE: Again, “SUN family” may be inaccurate. Thus, I changed “SUN family” to “SUN (Sad1p, UNC-84) domain proteins” (line 299). SUN (Sad1p, UNC-84) domain proteins localize to the INM. These proteins interact with Lamin and KASH (Klarsight, ANC-1 and SYNE1) domain proteins for nuclear positioning in a cell.

- Lines 271-272: Replace ‘domain’ with ‘luminal loop’

RE: The change has been made (line 300).

Reviewer 2 Advance summary and potential significance to field
The manuscript by Yamamoto-Hino reports that nuclear lamin B in Drosophila (lamin Dm0) is required for localisation of phosphatidylinositol glycan (PIG-B), an enzyme involved in the synthesis of glycosylphosphatidylinositol (GPI). This represents an important advance in the characterisation of GPI-related processes and a novel role to nuclear lamins. In a previous study, the authors found that failure ofPIG-B to localise to the nuclear envelope (NE) correlates with lethality in Drosophila. To understand themechanism by which PIG-B is recruited to the NE, the authors purified DIG-B-interacting proteins from S2 cells, ending up with a list of ~100 enriched proteins. Among these are Dm0 and lamin B receptor. The interaction of PIG-B with Dm0 is confirmed by reciprocal co-IP of endogenous proteins and by biotin proximity labelling. Depletion of Dm0 by RNAi or mutation prevents PIG-B NE accumulation. Conversely, by ectopic co-expression of Dm0 and PIG-B in CHO cells, the former is able to recruit the latter to the NE.
The interaction with Dm0 is specific in the sense that lamin C is not required for PIG-B localisation. Chimeras between lamins Dm0 and C demonstrates that the C-terminus of Dm0 is essential but not fully sufficient to recruit PIG-B. Importantly, the cellular distribution of GPI-anchored proteins is severely and similarly affected in Dm0 and PIG-B mutants.

Reviewer 2 Comments for the author
The manuscript is very well-written, clear and attractive. The quality of the experimental data seems very good and the authors discuss carefully their results in relation to the current status of the field. They propose several models for how PIG-B accumulates in the nucleus and why this is important. While the current data can stand on their own, I find it would strengthen that manuscript considerably if at least some of these possibilities were experimentally addressed. In particular, to determine if the interaction between Dm0 and PIG-B is required ‘only’ to position PIG-B at the NE or if Dm0 has an additional role in PIG-B activity, the authors could fuse PIG-B to an inner nuclear membrane protein (e.g. otefin) and test for rescue of GPI defects in Dm0 mutants.

RE: I appreciate the helpful comments. It is very difficult to generate functionally active chimeric proteins comprising DPIG-B and Otefin because the C-termini of DPIG-B and Otefin are localized within topologically distinct regions: the luminal side of the nuclear envelope and the nucleoplasm, respectively. Therefore, we tried to express Lamin CHRDT, which locates DPIG-B to the INM in LamK2 mutant flies. We then tried to examine whether GPI synthesis occurs normally in the INM in the absence of Lamin Dm0. However, such flies were lethal at the early developmental stage, thereby preventing analysis.
Specific points:

The data in Table S1 suggest that only a single control sample was used in the proteomics study. Please comment.

RE: Although the control experiment was performed only once, co-IP with DPIG-B revealed the concentration of nuclear and/or ER localized proteins. In addition, we have identified Lamin Dm0 as a protein required for NE localization of DPIG-B. Thus, we did not repeat this proteomic analysis.

© 2020. Published by The Company of Biologists under the terms of the Creative Commons Attribution License (https://creativecommons.org/licenses/by/4.0/).

I agree that the data presented are compatible with, and in favour of a model in which Dm0 and PIG-B interact directly and physically with each other, but definite evidence is lacking. All the interaction assays were performed in complex cellular contexts, thus, it cannot be ruled out that the interaction is indirect. I suggest this is commented in the manuscript.

RE: I agree. I have therefore worded the manuscript text more carefully.

In Figure 5 (and in the text), LamC is written as LamCHRCT. It would be simpler to maintain the name as LamC and only use the HR/T subscripts for the two chimeras.

RE: I have changed “Lamin C(HR)C(T)” to “Lamin C” to avoid confusion.

Phalloidin staining in the LamK2 mutant is more intense than in control flies, whereas the signal is weaker in the PIB-B13 mutant. Is this consistent and other samples and, if so, what could be the explanation?

RE: We recaptured the images of salivary glands from wild type, LamK2, and PIG-B13 mutants (Fig. 6A), and calculated the ratio of the FLAER signal at the plasma membrane to that in the cytoplasm (Fig. 6B). The results clearly show that FLAER signals in the cytosol were stronger in the LamK2 and PIG-B13 mutants.

Second decision letter

MS ID#: JOCES/2019/238527

MS TITLE: Lamin is essential for nuclear localization of the GPI synthesis enzyme PIG-B and GPI-AP production in Drosophila

AUTHORS: Miki Yamamoto-Hino, Kohei Kawaguchi, Masaya Ono, Kazuhiro Furukawa, and Satoshi Goto
ARTICLE TYPE: Research Article

I am happy to tell you that your manuscript has been accepted for publication in Journal of Cell Science, pending standard ethics checks. Thank you for your careful revisions.

© 2020. Published by The Company of Biologists under the terms of the Creative Commons Attribution License (https://creativecommons.org/licenses/by/4.0/).
